# Morbidity after conventional dissection of axillary lymph nodes in breast cancer patients

**DOI:** 10.1186/1477-7819-12-67

**Published:** 2014-03-27

**Authors:** Emerson Wander Silva Soares, Hildebrando Massahiro Nagai, Luis César Bredt, Ademar Dantas da Cunha, Reginaldo José Andrade, Géser Vinícius Silva Soares

**Affiliations:** 1 Department of Gynecology, Western Paraná State University (Universidade Estadual do Oeste do Paraná, UNIOESTE), Cascavel, Paraná, Brazil; 2 Department of Surgical Oncology, Study and Treatment Cancer Center of Western Paraná, (União Oeste Paranaense de Estudos e Combate ao Câncer, UOPECCAN), Cascavel, Paraná, Brazil; 3 Department of Surgical Oncology, UOPECCAN, Cascavel, Paraná, Brazil; 4 Department of Clinical Oncology, UOPECCAN, Cascavel, Paraná, Brazil; 5 Department of Radiotherapy, UOPECCAN, Cascavel, Paraná, Brazil

**Keywords:** Invasive breast cancer, Axillary lymph node metastases, Postsurgical complications, Lymphedema

## Abstract

**Background:**

Conventional axillary lymph node dissection (ALND) has recently become less radical. The treatment morbidity effects of reduced ALND aggressiveness are unknown. This article investigates the prevalence of the main complications of ALND: lymphedema, range-of-motion restriction, and arm paresthesia and pain.

**Methods:**

This cross-sectional study included 200 women with invasive breast cancer who underwent breast-conserving surgery (82.5%, n = 165) or mastectomy (17.5%, n = 35) with ALND from 2007 to 2011. Arm perimetry was used to assess lymphedema, defined as a difference >2 cm in the upper arm circumference between the nonsurgical and surgical arms. Range-of-motion restriction was assessed by evaluating the degree of arm abduction. Paresthesia was measured in the inner and proximal arm regions. Arm pain was assessed by directly questioning the patients and defined as either present or absent.

**Results:**

The average (±SD) time between ALND and morbidity evaluation was 35 ± 18 months (range, 7-60 months). The average dissected lymph node number per patient was 14 ± 4 (range, 6-30 lymph nodes). Only 3.5% (n = 7) of the patients presented with lymphedema. Single-incision approaches to breast tumor and ALND (*P* = 0.04) and the presence of a postoperative seroma (*P* = 0.02) were associated with lymphedema in univariate analysis. Paresthesia was the most frequent side effect observed (53% of patients, n = 106). This complication was associated with increased age (*P* < 0.0001) and a larger dissected lymph node number (*P* = 0.01) in univariate and multivariate analysis. Additionally, 24% (n = 48) of patients had noticeable limited arm abduction. Among the patients, 27.5% (n = 55) experienced sporadic arm pain corresponding to the surgically treated armpit. In multivariate analysis, the pain risk was 1.9-fold higher in patients who underwent ALND corresponding to their dominant arm (95% CI, 1.0-3.7, *P* = 0.04).

**Conclusion:**

Conventional ALND in breast cancer patients can result in unwanted complications. However, the current lymphedema prevalence is lower than that of the other analyzed side effects.

## Background

Axillary lymph node dissection (ALND), although controversial in specific situations, remains an integral part of surgical treatments in patients with invasive breast cancer and axillary lymph node metastases [1]. Specifically, this treatment is applicable in patients with tumors that are considered N1 or N2 according to the TNM staging system [2]. ALND was replaced in clinical practice by sentinel lymph node biopsy (SLNB) in patients that lack axillary lymph node involvement (N0) and some N1 patients [3], due to its reduced morbidity [4-7]. However, despite widespread mammography use for disease screening and early diagnosis, approximately one-third of patients in the U.S. suffer from tumors that have spread to the regional lymph nodes at diagnosis according to the Surveillance, Epidemiology and End Results (SEER) database [8]. In Brazil, according to National Department of Health statistics (2012), only 18% of these cancers are confined to the breast at diagnosis (pathologic stage), despite efforts to provide screening mammography for all women aged older than 40 years [9]. This late diagnosis means that, proportionally, more patients are submitted to ALND in Brazil than their American counterparts.

Currently, conventional ALND involves lymph node resection in levels I and II, as described by Berg [10]. Such dissections serve therapeutic functions and enable disease stage and prognosis assessments [11]. Unfortunately, ALND is primarily responsible for functional surgical treatment sequelae, including lymphedema, paresthesia, range-of-motion restriction, and pain in the arm ipsilateral to the lymph node dissection. Although esthetic sequelae that are caused by partial or total surgical breast resection can be reversed or minimized by reconstructive surgery methods that include prosthetics and tissue flaps, little can be done to correct the functional sequelae [12].

Since Halsted advocated the ‘systematic cleaning out of the axilla’ as an essential part of the operation ‘for the cure of cancer of the breast’ in 1907 [13], efforts to reduce the radicality and extent of tissue resection in ALND have been proposed. In 1948, Patey and Dyson [14] initially preserved the pectoralis major muscle but resected the pectoralis minor muscle along with ALND. Subsequently, Auschincloss [15] and Madden [16] proposed and closely described ALND with preservation of the pectoralis major and minor muscles in 1963 and 1965, respectively. The dissection of Berg level III has ceased in recent years [17], and the number of lymph nodes resected have decreased. A review of 21,992 women in the California Cancer Registry, who underwent ALND between 2004 and 2008, revealed an average of 11.4 ± 7.4 dissected lymph nodes per ALND [18]. In the 1960s, Auschincloss described an average of 38 lymph nodes per ALND [15]; this difference suggests that ALND has become increasingly conservative. Previous studies have shown that the incidence rates of complications and sequelae in the arm, including lymphedema, are directly related to the locoregional treatment radicality, which involves surgery and radiation therapy [19,20]. In the 1990s, studies showed that the incidence of lymphedema was decreasing due to more conservative approaches to the axilla [21].

Accordingly, this study aims to assess the current prevalence of ALND-associated complications in breast cancer patients.

## Methods

### Patient recruitment

A cross-sectional study was performed at the Cancer Study and Treatment Center of Western Paraná (União Oeste Paranaense de Estudos e Combate ao Câncer, UOPECCAN), a cancer patient treatment center in Cascavel city and a principal cancer treatment center in southern Brazil. The study was approved by the UOPECCAN Review Committee on Grant Proposals and Research Studies and the Research Ethics Committee of the State University of Western Paraná. Two hundred patients who underwent surgeries from January 2007 to December 2011 were evaluated. In all cases, informed consent and data collection was performed through specific interview and physical examination for the research.

### Inclusion and exclusion criteria

Women with histologically confirmed invasive breast carcinoma without distant metastases (M0) at diagnosis were included in this study. All patients underwent ALND associated with mastectomy or breast-conserving surgery and radiotherapy. Wherever possible, the ALND was performed by the same surgical incision used to approach the mammary tumor. When appropriate, the patients received chemotherapy, breast radiotherapy, immunotherapy, and hormonal blockade. No patient had the axillary nodal region included in the radiation field. The sequelae assessment was performed after chemotherapy or radiation therapy completion.

The exclusion criteria were defined as the presence of bilateral breast cancer, other malignancies except non-melanoma skin cancer and deformities, fractures, or previous surgery in the upper limb ipsilateral to the ALND. In the sequelae assessment period (July 2012 to December 2012), 33 potential candidates missed the study monitoring and were not located, 32 patients died from the disease or other causes, and four patients refused to participate.

### Research tools

1. Lymphedema (arm swelling)

The upper arm circumference (in cm) at 15 cm proximal to the lateral epicondyle ipsilateral to the axilla surgery site was compared with the contralateral upper arm circumference, just as described by Veronesi *et al*. [22]. Lymphedema was defined as a difference >2 cm in the upper arm circumference between the arm ipsilateral to the ALND and the non-surgical arm.

2. Paresthesia

The arm ipsilateral to the ALND was evaluated by touch response to a cotton ball on the inside of the arm at three points: proximal, medial, and distal; the resultant reaction was compared to that of the opposite arm. The patients were examined in a sitting position with the arms outstretched at 90 degrees. The evaluation result was defined as either present or absent (paresthesia).

3. Mobility

Rangeofmotion was evaluated by bilateral arm abduction with a protractor, and the categories were defined as follows: absent for 180 degrees of abduction, mild for 120 to 179 degrees of abduction, moderate for 90 to 119 degrees of abduction, and severe for abduction below 90 degrees.

4. Pain

The pain level was evaluated by directly asking the patient about the presence or absence of any arm pain. No pain score was used. The pain response was recorded as present or absent.

Additional data regarding patient characteristics, tumor stage, and treatment performed were obtained from medical records.

### Statistical analysis

The means and standard deviation (±) were used to assess numerical values. The associations between the variables studied (lymphedema, paresthesia, range-of-motion restriction, and pain) and the other patient characteristics were assessed using Fisher’s exact test for the binary nominal variables and the Mann-Whitney test for continuous numeric variables. Multivariate analysis was performed using multiple logistic regression tests for the binary variables and multiple linear regression tests for the continuous variables. A *P*value <0.05 was considered significant. The data were analyzed using the BioEstat® software statistical package, version 5.3(available at, http://www.mamiraua.org.br/).

## Results

The mean interval between ALND and analysis was 35 months (standard deviation, 18 months) with a range of 7 to 60 months. The average dissected lymph node number per patient was 14 ± 4 (range, 6-30 lymph nodes). None of the patients exhibited axillary recurrence during the analysis period. The patient and treatment characteristics are shown in Table 1.

**Table 1:** Clinical characteristics and treatments of the 200 patients in the study

**Characteristic**	**Number of patients (%)**
Age (years)	
Mean age	53 ± 11
<50	75 (37.5)
≥50	125 (62.5)
Dominant arm	
Right	188 (94)
Left	12 (6)
Surgery - evaluation interval	
Mean time (months)	35 ± 18
<36	112 (56)
≥36	88 (44)
Radiation therapy	
Yes	190 (95)
No	10 (5)
Chemotherapy	
Yes	192 (96)
No	8 (4)
Surgery	
Mastectomy	35 (17.5)
Conservative surgery	165 (82.5)
Dissected axilla	
Right	94 (47)
Left	106 (53)
Surgery in the dominant arm	
Yes	94 (47)
No	106 (53)
Incision for axillary dissection	
The same as the breast’s	122 (61)
Separate	78 (39)
Status of lymph nodes	
At least one metastatic	103 (51.5)
Absence of metastases	97 (48.5)
Lymph nodes with metastases (103)	
Mean positive lymph nodes	5 ± 5
<5	63 (61.1)
≥5	40 (38.8)
Dissected lymph nodes	
Mean dissected lymph nodes	14 ± 4
<10	35 (17.5)
≥10	165 (82.5)
Postoperative seroma	
Yes	20 (10)
No	180 (90)
Postoperative hematoma	
Yes	10 (5)
No	190 (95)
Postoperative infection	
Yes	9 (4.5)
No	191 (95.5)

Lymphedema was the least frequent complication and was found in only 3.5% (n = 7) of patients. Paresthesia was the most frequent complication and was observed in 53% (n = 106) of patients. Arm range-of-motion restriction, which was observed in 24% (n = 48) of the patients, and pain, which was reported by 27.5% (n = 55) of the interviewed patients, occurred with moderate frequency. Because 95% (n = 190) of the evaluated patients received breast radiotherapy and 96% (n = 192) received some type of chemotherapy to treat their breast cancer, both of these treatment modalities were excluded as potential variables from statistical analysis.

### Lymphedema

The low case number in this study precluded the multivariate analysis of lymphedema with the other variables. Associations between the presence of lymphedema with single-incision breast surgery (breast-conserving surgery or mastectomy) and ALND (*P* = 0.04) or with the presence of postoperative seroma (*P* = 0.02) were found via univariate analysis (Table 2).

**Table 2:** Relationships between lymphedema and the other variables

**Patient subgroups**	**Lymphedema**		***P *** **value**^**a**^
	**Yes (%)**	**No (%)**	
Mean age (years)	54 ± 12	53 ± 11	0.82
Surgery - evaluation interval (months)	34 ± 24	35 ± 17	0.84
Surgery			0.1
Mastectomy	3	32 (16.5)	
Conservative surgery	4	161 (83.5)	
ALND incision			0.04
The same as the breast’s	7	115 (59.5)	
Separate	0	78 (40.5)	
Axilla status			0.11
Positive	6	97 (50.2)	
Negative	1	96 (49.8)	
Surgery in the dominant arm			0.25
Yes	5	89 (46.1)	
No	2	104 (53.9)	
Positive axilla (103): number of positive lymph nodes	9 ± 10	5 ± 4	0.2
Dissected lymph nodes	16 ± 6	14 ± 4	0.36
Postoperative seroma			0.02
Yes	3	17 (8.8)	
No	4	176 (91.2)	

^a^Fisher’s exact test for binary nominal variables and the Mann-Whitney test for continuous numeric variables.

*ALND*: axillary lymph node dissection.

### Paresthesia

Paresthesia, the most frequent side effect of ALND, occurred mainly in older patients and in ALND patients with the most dissected nodes (Table 3). This association was observed in both the univariate and multivariate analyses. The average age of patients with paresthesia was 58 ± 11 years *versus* 49 ± 10 years for the group without paresthesia (*P* < 0.0001). On average, 15 ± 4 lymph nodes were dissected in the group with paresthesia *versus* 13 ± 4 lymph nodes in the group without paresthesia (*P* = 0.002).

**Table 3:** Relationships between paresthesia and the other variables

**Patient subgroups**	**Paresthesia**		***P *** **value**^**a**^
	**Yes**	**No**	
Mean age (years)	58 ± 11	49 ± 10	<0.0001
Surgery -evaluation interval (months)	34 ± 18	36 ± 18	0.32
Surgery			1
Mastectomy	19 (17.9)	16 (17)	
Conservative surgery	87 (82.1)	78 (83)	
ALND incision			0.56
The same as the breast’s	67 (63.2)	55 (58.5)	
Separate	39 (36.8)	39 (41.5)	
Surgery in the dominant arm			1
Yes	50 (47.2)	44 (46.8)	
No	56 (52.8)	50 (53.2)	
Axilla status			0.25
Positive	59 (55.7)	44 (46.8)	
Negative	47 (44.3)	50 (53.2)	
Positive axilla (103): number of positive lymph nodes	3 ± 5	4 ± 5	0.17
Dissected lymph nodes	15 ± 4	13 ± 4	0.002
Postoperative seroma			0.63
Yes	12 (11.3)	8 (8.5)	
No	94 (88.7)	86 (91.5)	

^a^Fisher’s exact test for binary nominal variables and the Mann-Whitney test for continuous numeric variables.

*ALND*: axillary lymph node dissection.

### Range-of-motion restriction

Although range-of-motion restriction, as assessed by the degree of abduction of the arm ipsilateral to ALND, was found in 24% (n = 48) of patients, it was considered mild in 15% (n = 30), moderate in 8% (n = 16), and severe in only 1% (n = 2) of patients. The univariate analysis revealed an association between the range-of-motion restriction with single-incision breast surgery (breast-conserving surgery or mastectomy) and ALND (*P* = 0.02, Table 4). However, this association was not confirmed by the multivariate analysis.

**Table 4:** Relationships between range-of-motion restriction and the other variables

**Patient subgroups**	**Range-of-motion restriction**		***P *** **value**^**a**^
	**Yes**	**No**	
Mean age (years)	54 ± 12	53 ± 11	0.96
Surgery -evaluation interval (months)	35 ± 20	35 ± 17	0.75
Surgery			0.12
Mastectomy	12 (25)	23 (15.1)	
Conservative surgery	36 (75)	129 (84.9)	
ALND incision			0.02
The same as the breast’s	36 (75)	86 (56.6)	
Separate	12 (25)	66 (43.4)	
Surgery in the dominant arm			0.6
Yes	24 (50)	70 (46.1)	
No	24 (50)	82 (53.9)	
Axilla status			0.74
Positive	26 (54.2)	77 (50.6)	
Negative	22 (45.8)	75 (49.4)	
Positive axilla (103): number of positive lymph nodes	7 ± 6	4 ± 4	0.1
Dissected lymph nodes	14 ± 5	14 ± 4	0.89
Postoperative seroma			0.78
Yes	4 (8.3)	16 (10.5)	
No	44 (91.7)	136 (89.5)	

^a^Fisher’s exact test for binary nominal variables and the Mann-Whitney test for continuous numeric variables.

*ALND*: axillary lymph node dissection.

### Pain

Sporadic pain in the arm ipsilateral to the ALND, which was present in 27.5% (n = 55) of the evaluated patients, did not associate with the other variables in a univariate analysis. However, the pain risk was 1.9-fold higher in patients who underwent ALND ipsilateral to the dominant arm (95% CI, 1.0-3.7; *P* = 0.04), according to the multivariate analysis (Table 5).

**Table 5:** Relationships between pain and the other variables

**Variables**	**Odds ratio**	**95% CI**	***P-*** **value**^**a**^
Mean age (52 years *vs.* 54 years)^b^			0.16
Surgery – Evaluation. interval (34 *vs*. 36 months)^b^			0.41
Surgery (mastectomy*vs.* conservative breast surgery)	1.4	0.6-3.5	0.38
Incision (single *vs.* separate)	0.9	0.4-2.0	0.95
Surgery (dominant arm *vs.* non-dominant arm.)	1.9	1.0-3.7	0.04
Axilla status (positive *vs.* negative)	1.3	0.7-2.6	0.37
Number of positive lymph nodes (6 *vs.* 5)^b^			0.24
Number of dissected lymph nodes (14 *vs.* 14)^b^			0.65
Postoperative seroma (yes *vs.* no)	0.7	0.2-2.3	0.65

^a^Multivariate analysis: multiple logistic regression tests for binary variables and multiple linear regression for continuous variables.

^b^Multiple linear regression for continuous variables does not generate odds ratio and CI.

*CI*: confidence interval.

## Discussion

ALND’s importance in the staging, prognostic assessment and local control of breast cancer has long been proven [23]. Local control of breast cancer also results in longer survival [24], and failing to ‘clean’ the axilla could mean a loss of disease control with distant metastases, such as that noted in the NSABP B-04 study [25]. If not for the morbidity, the need for ALND would not be debated in cases of SLNB positivity [3]. In contrast, the incorporation of SLNB in breast cancer treatment has benefited many women with the disease at an early stage, confined to the breast, avoiding ALND. This treatment course substantially decreased functional sequelae risks in the arm that result from surgical armpit manipulation. Because women are increasingly surviving breast cancer [26], ALND morbidity is generating a high social, psychological, and financial cost.

Unfortunately, 40.4% of patients in Brazil already present with stage III and IV disease at diagnosis [9]. In this study, 51.5% of the patients (n = 103) had metastases in the axillary lymph nodes. The N0 patients who underwent ALND (48.5%, n = 97) had contraindications to SLNB (for example, inflammatory breast cancer, T4 tumors, clinically positive axilla) or refused the procedure.

Currently, conservative surgery combined with breast radiation therapy is considered as effective as total mastectomy for the local control of breast cancer [27,28]. This combination treatment was administered to 82.5% (n = 165) of the patients in this study. Previous studies have shown that the type of treatment used influences the morbidity prevalence. Schünemann and Willich [19] evaluated 5,868 patients with breast cancer treated from 1972 to 1995 and demonstrated that the addition of radiotherapy to modified radical mastectomy increased the lymphedema incidence from 19.1% to 28.9%. DiSipio *et al*. [20], in a systematic review and meta-analysis that evaluated 72 studies, associated chemotherapy with lymphedema. These two variables (radiotherapy and chemotherapy) were not evaluated in this study, because the vast majority of the patient cohort (95% and 96% respectively) was subjected to these therapeutic modalities.

Lymphedema is by far the most serious and difficult to treat complication that has the greatest effect on a patient’s quality of life. However, lymphedema is not the only ALND-related complication. The literature primarily reports lymphedema, paresthesia, pain, and range-of-motion restriction as complications of ALND. The risk of complications correlates positively with the radical nature of ALND [19,29].

The lymphedema incidence and prevalence described in the literature vary widely, possibly due to different measurement methods and intervals between ALND and lymphedema measurement. DiSipio *et al.*[20] observed a 21.4% lymphedema incidence after analyzing 30 prospective cohort studies. Therefore, the 3.5% prevalence observed in this study is below the literature average. The lymphedema measurement method used in this study (circumference measurement) is simple, easily reproducible, and the most used approach in the medical literature. Using this method, the average lymphedema incidence observed was 14.8%, as described by DiSipio *et al.*[20] Another study using the same lymphedema assessment criteria (upper limb measurement difference of >2 cm between the arms) was conducted in Greece by Keramopoulos *et al.*[30] and reported a 17% lymphedema incidence. Possible explanations for this unexpectedly low lymphedema prevalence are the lack of systematic dissection of level III, the lack of interpectoral space exploration (Rotter lymph nodes), and the attempted preservation of the pectoral muscles. Evidently, this approach results in fewer dissected lymph nodes, as noted in the present study with an average of 14 dissected lymph nodes per patient. However, none of the patients showed axillary recurrence during the analysis period. Although this number is lower than the 38 dissected lymph nodes per patient reported in the 1960s [15], 10 dissected lymph nodes per patient is currently considered to be an adequate minimum number [31,32]. It is also important to note that the time between ALND and analysis was less than 2 years in some patients. The review conducted by DiSipio *et al*. [20] demonstrated that lymphedema appears to increase 2 years after diagnosis of or surgery for breast cancer. The fact that no patient had the axillary nodal region included in the radiation field may also have contributed to reduce the risk of lymphedema.

Finally, another important aspect to be considered is that difference of >2 cm between the arms may be too much to objectively evaluate lymphedema morbidity and an investigation with upper limb lymphoscintigraphy, for example, would have helped to detect lymphatic impairment much earlier than clinically. Therefore, this may have led to an underestimation of lymphedema prevalence in our results.

Paresthesia was the most frequent complication in this study and was found in more than half of the patients (53%, n = 106). Veronesi and colleagues [22] reported a prevalence of 68%, and Warmuth *et al.*[12] reported a prevalence of 35%. This finding is related to the intercostobrachial nerve section that crosses the axilla and is transected during ALND. However, paresthesia is a minor complication that neither results in complaints nor limits the quality of life in most cases [33]. There was no intent to preserve the intercostobrachial nerve (ICBN) during ALND for the patients evaluated in the present study.

Pain and range-of-motion restriction occurred in 27.5% (n = 55) and 24% (n = 48) of the patients, respectively. The range-of-motion restriction was evaluated according to the degree of arm abduction, a method that has been previously used [34]. The pain and range-of-motion restriction incidence rates vary widely in literature reports. Warmuth *et al.*[12] identified an 8% limitation in arm movement and a 30% incidence of pain in 432 patients 2 to 5 years after ALND. Kootstra *et al.*[35] evaluated 76 women and observed that 70% had clinical relevant impairments in the shoulder and arm 7 years after ALND. It is noteworthy that 62.5% (n = 30) of patients who presented with range-of-motion restriction (n = 48) had only mild range-of-motion restriction (120° to 179° abduction). The range-of-motion restriction might be overestimated in the present study because a rangeofmotion of at least 160° can be considered normal [36].

This study also found a correlation between lymphedema and single-incision surgery. This fact was not addressed in related previous studies. A possible explanation is that in breast-conserving surgery, attempts to approach the breast (quadrantectomy) and axilla (ALND) through the same incision sometimes create tunnels that complicate the correct identification of planes that involve the axilla and the preservation of the periaxillary subcutaneous tissue.

The present study also found an association between paresthesia and advanced patient age. Ververs and colleagues reported an opposite finding [37]. They discovered an inverse relationship between arm paresthesia and patient age.

The present study showed that pain, a subjective datum, was associated with surgery ipsilateral to the dominant arm. This association was also found in a previous study by Hayes and colleagues [38].

## Conclusion

In conclusion, although SLNB has benefited many women with breast cancer, many patients still require ALND, despite its associated risk for morbidity. The results of this study showed that the lymphedema prevalence, the most undesirable ALND side effect, was low in relation to other evaluated symptoms. Further, larger prospective studies are required to fully assess the exact morbidity incidence following ALND.

## Competing interests

The authors declare that they have no competing interests.

## Authors’ contributions

EWSS conceived of the study, participated in its design and coordination, analysis and interpretation of data and helped to draft the manuscript. HMN participated in acquisition of data, have been involved in the design of the study and performed the statistical analysis. LCB participated in acquisition of data and have been involved in statistical analysis. ADCJr participated in its design and coordination and helped to draft the manuscript. RJA participated in its design and coordination and helped to revising the manuscript. GVSS participated in its design and coordination and helped to revising the manuscript. All authors read and approved the final manuscript.
